# Longitudinal CT Scanning for Explainable Early Detection of Postharvest Disorders: The ‘Braeburn’ Browning Case

**DOI:** 10.3390/jimaging12070331

**Published:** 2026-07-21

**Authors:** Dirk Elias Schut, Rachael Maree Wood, Rob Schouten, Robert van Liere, Tristan van Leeuwen, Kees Joost Batenburg

**Affiliations:** 1 Computational Imaging Group, Centrum Wiskunde en Informatica (CWI), 1098 XG Amsterdam, The Netherlands; 2 Horticulture and Product Physiology, Wageningen University and Research, 6708 PB Wageningen, The Netherlands; 3 Plant Production Systems Group, Wageningen University, 6708 PE Wageningen, The Netherlands; 4 Wageningen Food and Biobased Research, 6708 WG Wageningen, The Netherlands; 5 Visualization Cluster, Eindhoven University of Technology, 5612 AP Eindhoven, The Netherlands; 6 Mathematisch Instituut, Utrecht University, 3584 CS Utrecht, The Netherlands; 7 Leiden Institute of Advanced Computer Science (LIACS), Leiden University, 2333 CC Leiden, The Netherlands

**Keywords:** non-destructive testing (NDT), deep learning, explainable artificial intelligence (XAI), image registration, Integrated Gradients, postharvest, Apple, internal browning

## Abstract

This study presents two workflows for leveraging longitudinal computed tomography (CT) datasets when developing deep learning-based detection systems for gradually developing postharvest disorders. Workflow 1 (Longitudinal Benchmarking) benchmarks neural networks by training and testing them on images from different stages of disorder progression. It examines the trade-off between detecting a disorder early or accurately and evaluates whether neural networks can generalize across time points. Workflow 2 (Longitudinal eXplainable Artificial Intelligence (XAI) Heatmaps) provides heatmaps that indicate how changes over time affect the outcomes of neural networks. It uses image registration to align an earlier-acquired image and then uses it as a baseline when calculating the heatmap. The workflows are demonstrated on a dataset of ‘Braeburn’ apples that were CT-scanned multiple times while developing internal browning during controlled-atmosphere (CA) storage and shelf life. The Longitudinal Benchmarking workflow was used to investigate whether images acquired immediately after CA storage can be used to predict the eventual browning after a shelf-life period, which is highly relevant in industrial practice. Moreover, the longitudinal XAI heatmaps avoided artifacts caused by out-of-distribution baselines or identical baseline regions, which occurred with conventional black or zero baselines.

## 1. Introduction

Roughly one-third of the food produced for human consumption is wasted globally [1]. Sorting agricultural products based on non-destructive measurements can reduce waste and improve customer satisfaction [2]. Photography is a commonly used modality for postharvest disorder detection. However, it can only detect disorders that are visible on the product’s exterior. To assess the internal quality of a product, X-ray imaging can be used [3]. The two main X-ray imaging methods are radiography, which is faster and cheaper, and computed tomography (CT), which provides higher contrast and enables 3D imaging. Image classification algorithms can automatically assign a grade to each product on a sorting line based on the acquired images. Deep learning is a state-of-the-art technique for image classification, and it has been successfully applied for detecting disorders from CT or radiography images of many crops, such as apples [4], pears [5,6], avocado [7], and mango [8].

One challenge in detecting postharvest disorders is that many of them develop gradually. They may be difficult to detect at an early stage because they may not yet be clearly visible on the images provided to the neural network. However, detecting a disorder at an early stage may also be valuable, for example, to limit the spread of infectious disorders, such as fruit flies [9]. Moreover, some foods are still consumable if a disorder is detected at an early stage, but not at a later stage, such as apples with bitter pit [10], or pears with mealiness [11]. To study disorder progression, multiple scans over time may be acquired of the same samples, forming a longitudinal dataset. Longitudinal CT datasets have been acquired to study several postharvest processes, such as internal browning in apples [12,13], bitter pit in apples [10], mealiness in pears [11], the preservation of strawberries and persimmon using edible coatings [14,15], and the aging of cucumbers [16]. However, to the knowledge of the authors, there is no prior work on deep learning-based classification of postharvest disorders that accounts for their progression over time.

Another challenge is that the decision-making of deep learning-based image classification networks is often unclear. The weights of the neural network after training fully specify the network’s behavior, but they are impossible for humans to interpret directly due to the large number of weights and their complex structure. Explainable artificial intelligence (XAI) heatmaps (also called attribution maps or saliency maps) aim to provide a more easily understandable overview of a neural network’s decision-making process. For a given neural network and input image, they show how much each region contributed to the network’s decision [17,18,19,20]. However, there are many methods for calculating XAI heatmaps with strongly different results [21], and it can be unclear what a heatmap represents [22].

**Figure 1:**
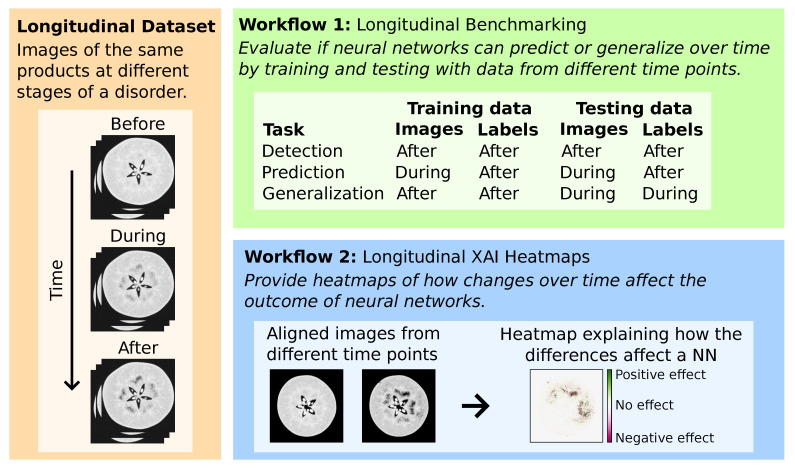
Illustration of the two workflows in this paper.

The key contribution of this paper is that we provide two workflows for using longitudinal imaging datasets in the development of deep learning-based image classification systems for postharvest disorders, which are both illustrated in Figure 1:

- Workflow 1: Longitudinal Benchmarking
- Workflow 2: Longitudinal XAI Heatmaps

The workflows only use longitudinal data during the development of classification systems. When the system is deployed, each product is scanned only once, and the image can be processed immediately, which is more practical for high-throughput industrial applications. Workflow 1 benchmarks neural networks by training and testing them on images from different stages of disorder progression. This makes it possible to investigate how much early-stage disorder detection affects accuracy and whether classification networks trained on data from one time point generalize to data from other time points. Workflow 2 provides heatmaps of how changes over time affect the outcome of neural networks. It avoids common artifacts of other XAI methods by only considering the differences between the input image and an earlier image of the same object.

The problem of detecting internal browning in apples of the ‘Braeburn’ cultivar is used as an example to demonstrate the workflows. We use a dataset from previous research [12,23], in which apples were CT scanned before, during, and after controlled-atmosphere (CA) storage, and afterwards scored for browning on a 1–4 scale. Radiographs can be simulated from CT scans [24], so we use this CT dataset to demonstrate the workflows on CT and radiography.

## 2. Related Work

### 2.1. Internal Browning in Apples

Internal browning (IB) is a disorder in apples and pears that is characterised by brown regions inside the fruit with an unpleasant taste. IB develops during controlled-atmosphere (CA) storage, which is used to store apples or pears for long periods to supply markets with a high-quality product year-round [25]. Browning occurs when cellular integrity breaks down, leading to the oxidation of phenolic compounds [26]. During this process, the affected tissues can also leak their intracellular fluids, which can then move towards the surrounding healthy tissues [27]. These changes in fluid distribution are visible on X-ray images due to the resulting differences in density. Regions with browning typically have a lower density, while surrounding tissues may have a higher density [27,28]. Depending on the apple cultivar, storage conditions, and several other pre- and post-harvest factors, different patterns of browning may occur [23,28]. Apples of the ‘Braeburn’ cultivar are especially susceptible to core browning [29,30], which affects the tissue around the core of the apple while the region closer to the peel remains unaffected, making non-destructive visual detection of this disorder difficult.

### 2.2. Postharvest Longitudinal CT Scanning

In the postharvest domain, longitudinal CT studies have been performed to study the progression of disorders [10,11,12,13,31,32,33], the spread of induced bacterial, fungal, or insect infections [9,34], aging [16,35], and the effects of coatings on long-term storage [14,15,35]. These datasets were investigated visually, or quantitative metrics were derived based on manual annotations or image processing techniques. Most studies scanned the whole fruit or vegetable, but some studies used high-resolution micro-CT scans of a small sample [36,37]. In several studies [11,13,31,36,37], scans of the same product were spatially aligned using image registration methods [38] to enable more precise comparisons between images or further processing.

On the topic of internal browning, a longitudinal CT dataset of pears was used to study internal browning and to model the gas transport inside the fruit [31]. A paper on detecting disorders by combining information from radiographs and 3D surface scans collected a longitudinal CT dataset of apples as a reference dataset [13]. That dataset does not contain annotations or labels for specific disorders, but the paper shows an apple that develops core browning as an example. An earlier study from our research project investigated a longitudinal CT dataset of ‘Braeburn’ apples with browning labels to investigate how browning progresses before, during, and after CA storage [12]. That study found that the CT scans of apples that eventually would develop internal browning had a lower mean grey value at about 12 weeks of CA storage at a significance of *p* < 0.1. These differences became more significant (*p* < 0.05) when the CA storage was 18 weeks, or when a shelf-life period was added after CA storage. The experiments in this paper use the same dataset and extend the previous study by classifying individual apples using deep learning. To the authors’ knowledge, this is the first study to apply deep learning to a longitudinal postharvest CT dataset.

### 2.3. AI-Based Postharvest Disorder Detection

A recently written introduction paper provides an overview of the research on using artificial intelligence for detecting postharvest disorders [39]. Image classification neural networks trained on radiography or CT datasets have been used to detect various types of postharvest disorders [4,5,6,7,8]. Deep learning-based image segmentation [40], object detection [41], and outlier detection [42] approaches have also been used.

To detect internal browning in ‘Braeburn’ apples, Tempelaere et al. [4] developed deep learning-based classifiers for CT scans and radiographs, and they achieved more than 95% accuracy on both types of data. However, they used different CA conditions for the apples that were labeled as brown or healthy, and they stored the apples in shelf-life conditions for 3 days before CT scanning, which together resulted in relatively large differences between the brown and healthy apples. In our dataset, the same storage conditions were used for all apples, and in the prediction task from Workflow 1 (Section 3.2), the apples are classified based on data from the day the apples were removed from CA storage. This is more in line with industrial practice, in which all apples are stored in the same CA conditions, sorted shortly after leaving CA storage, and consumed after a shelf-life period.

### 2.4. Explainable AI (XAI) Heatmaps

XAI heatmaps can be calculated in many ways, for example, by using gradients [17,19,43,44], internal activations of the network [20,45], local surrogate models [46], input perturbations [47,48,49], and propagating scores through the network back to the inputs [50,51]. Heatmaps calculated by different methods on the same image can vary a lot [21], and users commonly misinterpret heatmaps in practice [22].

To more precisely specify what heatmaps should represent, Sundararajan et al. [17] introduced the concept of axiomatic heatmap methods. Axiomatic heatmap methods are designed to satisfy appealing axioms (guaranteed mathematical properties), such as retaining linearity or symmetry of the neural network. Sundararajan et al. also introduced Integrated Gradients (IG) as the first axiomatic method [17], and the axioms of IG have been further investigated [18]. Several other XAI methods have been developed that satisfy many of the same axioms as IG [19,44,47,48].

One challenge in using IG and many other axiomatic XAI heatmap methods is that they require a baseline image as a second input. This baseline image is used as a neutral reference point in many of the axioms. The baseline image has a high impact on the result [52], and it may be challenging to find a suitable neutral baseline for a given problem [53,54]. In Workflow 2 (Section 3.3), we propose using an image from an earlier time point as a baseline, where we assume that in this image, the disorder is not yet present or less developed. This way, the baseline represents an object-specific neutral state instead of a general neutral state. Mamalakis et al. [54] also used measurements from an earlier time point as a baseline to explain changes over time in a global climate model. Our Workflow 2 (Section 3.3) is similar, but it extends that work by using image registration to align the baseline with the input. Moreover, we investigate how this approach can be used to avoid artifacts related to out-of-distribution data [19], and regions that are the same in the baseline and input [52,53].

Previous research in postharvest disorder detection has used different XAI heatmap methods. The GradCAM method [20], used in [4,55,56], has a very low resolution because of how it is calculated. The LIME method [46], used in [57], only explains the behavior of the neural network in a region around the input. Moreover, it assigns the scores to superpixels (regions from a segmentation step), instead of to individual pixels, which reduces visual noise but limits the effective resolution. Workflow 2 provides full-resolution heatmaps with a well-defined mathematical foundation that explain how the neural network interpreted all changes between the baseline and input images.

## 3. Materials and Methods

### 3.1. Data Acquisition

Both workflows require a longitudinal dataset of images. How the longitudinal ‘Braeburn’ browning dataset [12,23] was collected and reconstructed is described below.

#### 3.1.1. Apple Samples and Browning Score

In 2022, 80 ‘Braeburn’ apple fruit were harvested at the optimal harvest date from the Randwijk experimental orchard, The Netherlands. To cause internal browning to develop, the apples were stored in controlled-atmosphere (CA) storage under non-optimal conditions (0.5 °C, 1.5 kPa O_2_, 5 kPa CO_2_). The apples were stored in CA storage for 0, 3, 9, 12, or 17 weeks. After CA storage, the apples were stored in shelf-life conditions (19–20 °C) for up to three weeks. Varying durations of CA and shelf-life storage were used to ensure variety in the browning severity. Moreover, due to limited scanner availability and long scanning times, not all apples were scanned at each interval. After the last CT scan, the fruit were sliced with a knife into approximately 10 mm slices, photographed, and scored for browning intensity. Browning intensity was scored based on visual inspection of the slices on a subjective scale from 1 to 4, where 1 indicates no brown tissue and 2, 3, and 4 indicate mild, moderate, and severe browning, respectively. One apple was excluded because it developed severe rot.

#### 3.1.2. CT Scanning Protocol

The apples were scanned at the FleX-ray laboratory using a custom scanner developed by TESCAN-XRE, Gent, Belgium. A cone beam geometry with a circular trajectory was used to acquire 1440 projection images at an exposure time of 100 ms, a tube peak voltage of 90 kV, and a current of 550 μA. A detector pixel binning of two was used, and the voxel size was between 130.6 μm and 134.3 μm. The voxel sizes differed slightly between scans due to one scanner motor being defective on some scan days, which limited the range of possible resolutions but otherwise did not affect the scans. The average time per scan was five minutes.

#### 3.1.3. Image Reconstruction

The FDK algorithm [58] was used for reconstructing the CT volumes. Third-order polynomial beam hardening correction was used [59], using a paper cup filled with apple juice as a calibration phantom. Gradient descent with momentum was used to optimize the beam hardening parameters [60] by using the differentiable FDK implementation from the Tomosipo library [24].

### 3.2. Workflow 1: Longitudinal Benchmarking

#### 3.2.1. Conceptual Overview of the Workflow

This workflow evaluates how much the performance of image classification networks is affected by the progression of the disorder. We distinguish three main tasks. For each task, neural networks are trained and tested on different combinations of input images and ground truth labels. **Detection**: Training and testing use images and labels from the same time point. This is the standard scenario for detection problems, and this does not require a longitudinal dataset. **Prediction**: Training and testing use images from one time point, but labels from a later time point, so the network should predict a future state of the product. **Generalization**: Networks are tested on data from a different time point than they were trained on.

#### 3.2.2. Detection, Prediction and Generalization on the ‘Braeburn’ Browning Dataset

Four networks were trained to demonstrate the workflow on the ‘Braeburn’ browning dataset. The input data modality was either CT slices or radiographs. For both modalities, one network was trained to detect the browning score after shelf-life, and another was trained to predict the future browning score after CA storage but before shelf-life. Only apples that had been in CA storage for at least nine weeks were included. For the prediction task, only apples that were sliced after two weeks of shelf life were included. An overview of which apples were used for the detection and prediction tasks is provided in Table 1.

To evaluate the networks’ ability to generalize to data from different time points, the detection networks were also tested on the data of the prediction task and vice versa.

#### 3.2.3. Neural Network Architecture

The network architecture was an EfficientNetV2_s [61] using the implementation from torchvision [62] for all networks. This network architecture was chosen because it has shown a good performance relative to the training time and network size when compared to other networks [61]. The networks were converted for grayscale inputs by replacing the original three input channels (RGB-color) with one grayscale input channel. Moreover, the networks were converted to regression networks by replacing the linear layer and the softmax activation function at the output side of the networks with a single-output linear layer. By converting the networks into regression networks, the browning score was represented as a single continuous variable rather than four distinct classes. This more accurately reflects the ordered nature of the browning scores. Moreover, it benefits the calculation of XAI heatmaps in Workflow 2.

#### 3.2.4. Usage of the Dataset

A 60%, 20%, 20% split was used between the training, validation, and test sets. The exact number of images used is given in Table 2. Stratified sampling ensured each set contained a similar ratio of apples from each day and with each browning label. For the test set, if possible, apples were selected for which a before-storage scan was available, which was required for Workflow 2 (Section 3.3). Data augmentations were applied using the Albumentations library [63] and the images were normalized based on the mean and standard deviation of the training set.

For the networks trained on the CT slices, in each epoch, one horizontal slice was randomly selected from the region 10% above or below the center of mass of the core of the apple, resulting, on average, in 123 slices being available per apple. The data augmentation consisted of random rotations, flipping, shearing, translations, scaling, and elastic deformations [64].

The radiography neural networks were trained on the radiographs that were also used as the raw measurement data for reconstructing the CT scans. Flatfielding and the log-transformation were applied as preprocessing steps. To reduce storage space, only every third preprocessed radiograph was stored, resulting in 480 radiographs per apple. In each epoch, one radiograph was randomly selected per apple. The data augmentation consisted of random rotations, horizontal flipping, shearing, scaling, and elastic deformations [64]. Moreover, roughly 20% of the top and bottom of the radiograph were cropped to remove parts where browning does not occur.

#### 3.2.5. Neural Network Training

The neural network training was implemented using Pytorch (version 2.2.0) [65] and Pytorch Lightning [66]. The cost function was the mean absolute error (MAE). The optimizer was ADAMW [67], with learning rates of 1 × 10^−5^ and 2 × 10^−5^, weight decays of 0.05 and 0.13, and batch sizes of 20 and 22 for the CT and radiography networks, respectively. No learning rate scheduling was used. The training duration was approximately 20,000 epochs (2 days on 2 RTX Titan GPUs). At the end of each epoch, the MAE was calculated on the validation set. The network weights with the lowest validation MAE were used in the Section 4.

#### 3.2.6. Evaluation Metrics

To evaluate the neural networks, two metrics were calculated. For both metrics, the continuous neural network output was converted to a browning score by rounding to the nearest integer and then clipping to the 1–4 range. The first metric was the MAE of the browning score after rounding and clipping. The second metric is the percentage of apples correctly classified as brown or not, where browning scores 2, 3, and 4 are considered brown, and score 1 is considered healthy. This second metric was included for easier comparison with other papers that do not consider multiple levels of browning.

For each apple, multiple CT and radiography images were available, but the neural networks could return different scores for each image. The median score per apple was used to reduce errors and improve browning predictions. In the results, we report both per-image and per-apple scores.

### 3.3. Workflow 2: Longitudinal XAI Heatmaps

#### 3.3.1. Conceptual Overview of the Workflow

This workflow provides heatmaps of how changes over time affect the outcome of neural networks. It works by applying any existing XAI heatmap method that satisfies the Completeness axiom while using an earlier image of the same product as the baseline, which we will call a longitudinal baseline. Image registration is used to spatially align the longitudinal baseline to the input. Using a longitudinal baseline is likely to avoid common issues, where the baseline is out-of-distribution or where the baseline is equal to the input image in important regions.

#### 3.3.2. The Integrated Gradient (IG) Method

We demonstrated the workflow using the Integrated Gradients (IG) method [17]. For a given neural network *F*, input image $\bar{x}$ and baseline image $x^{\prime }$ the IG heatmap $A^{\mathrm{IG}}\left(\bar{x},x^{\prime },F\right)$ was calculated as follows:

(1) $$A^{\mathrm{IG}}\left(\bar{x},x^{\prime },F\right)=\left(\bar{x}-x^{\prime }\right)\int_{\zeta =0}^{1}\nabla F\left(\left(1-\zeta \right)x^{\prime }+\zeta \bar{x}\right)d\zeta .$$

IG satisfies the Completeness axiom. This axiom guarantees that the sum over all pixels of the heatmap is equal to the difference in neural network output between the input and the baseline:

(2) $$\sum_{i=1}^{n}A_{i}^{\mathrm{IG}}\left(\bar{x},x^{\prime },F\right)=F\left(\bar{x}\right)-F\left(x^{\prime }\right).$$

By using an image that was acquired at an earlier time point as the baseline, the heatmap shows how much the changes over time affect the neural network’s outcome.

#### 3.3.3. Explaining the ‘Braeburn’ Browning Networks

To demonstrate the workflow on the ‘Braeburn’ browning dataset, we calculated heatmaps on how much each part of the image contributed to the browning score. We used the before-storage data as a longitudinal baseline, which should be unaffected by browning, as browning develops during storage. For the CT networks, the corresponding slice from the registered before-storage CT scan was used. For the radiography networks, a corresponding radiograph was simulated from the registered CT scan.

#### 3.3.4. Image Registration

All CT scans were aligned in 3D to the first available CT scan of the same apple by using image registration with the SimpleITK library [68,69]. All apples were scanned with the stem on top, but the rotation around the stem–calyx axis differed between scans. To avoid converging to the wrong local minimum, the first optimization step of the registration method was performed ten times with a different initial rotation. The initial rotations were equally spaced between 0 and 360 degrees around the vertical axis. In this first step, a rigid transformation model was optimized over the MSE using the L-BFGS optimizer [70] with four and two times downsampling. The image registration parameters with the lowest MSE were used as initialization for the second step. In the second step, a similarity transformation model was optimized over the MSE using the regular step gradient descent optimizer with four times, two times, and no downsampling.

#### 3.3.5. Evaluating the Longitudinal Baseline

The workflow was evaluated on the ‘Braeburn’ browning dataset by comparing the longitudinal baseline with two other common baseline choices: the constant black baseline (zero before normalization), which was suggested in the original IG paper [17], and the constant zero baseline (zero after normalization), which is the default of the Captum XAI library [71].

One property that a baseline should have is that it has a neutral output [17]. On the networks trained for browning detection, a neutral output is a browning score of one, so $F(x^{\prime })=1$. In that case, using the Completeness axiom (Equation (2)), the value of each pixel of the heatmap ($A_{i}^{IG}$) can be interpreted as how much that pixel contributed towards increasing the browning score from 1. When the baseline output has a large value, the heatmap will mostly explain the baseline and not the input. Therefore, the output values of the neural network on the considered baseline options were calculated, and they should be close to 1. Out-of-distribution data points may have high output values because the network was not optimized for these points during training.

Another property that a baseline should have is that it should not be equal to the input image in regions that correspond to the disorder. Due to how IG heatmaps are calculated, they are guaranteed to have a value of zero for pixels that are equal between the baseline and the input (${\bar{x}}_{i}=x_{i}^{\prime }$). This makes IG heatmaps blind to these regions [52,53]. Regions of severe browning may show up as black spots on a CT scan [28], so IG with a black baseline is blind to these regions, which is undesirable when trying to explain what contributes to a browning score. IG with a longitudinal baseline is blind to parts of the image that did not change over time. If you assume that apples are completely free of browning before storage, this is not a problem. The heatmaps were visually inspected for regions where they were equal to their inputs, and the heatmaps were checked for blind spots.

## 4. Results

### 4.1. Workflow 1: Longitudinal Benchmarking

The results of the neural networks on the test set are shown in Table 3. The neural networks trained on CT slices had better MAE and accuracy scores than the networks trained on radiographs in all cases. Combining the outputs from multiple input images of the same apple also improved results in all cases, resulting in 100% accuracy in the detection and prediction of browning from CT slices.

To put these results into context, consider a classifier that always predicts the same score. The score yielding the best results would be 2, with an MAE of 0.9333 on the detection task and 1.0 on the prediction task, and brown/healthy accuracies of 66.7% and 60%, respectively. All four neural networks strongly outperform such a classifier, indicating that they successfully learned to extract relevant information from the image data.

Table 4 shows the per-image MAE when the detection neural networks were applied to the prediction data and vice versa. It shows that the neural networks did not generalize across time points. Instead, they classified most apples at one side of the score range. The detection networks on the just-out-of-storage data classify most apples as healthy (score 1), while the prediction networks on the after-shelf-life data classify most apples as severely brown (score 4).

### 4.2. Workflow 2: Longitudinal XAI Heatmaps

**Figure 2:**
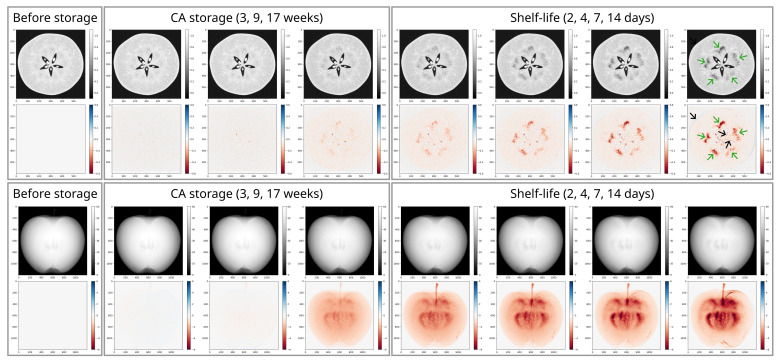
(**Top**) Central slice of registered CT scans over time, and difference images showing the difference from the before storage scan. (**Bottom**) Simulated radiographs of the same apple over time, and difference images showing the difference from the before-storage radiograph. The images show that browning develops both during CA storage and shelf life. The regions associated with browning are marked with green arrows in the last CT slice and difference image. When there is a registration error, it results in registration artifacts in the difference images around the edges of the apple or the apple core. These artifacts are marked with black arrows in the last CT difference image (you may only see them clearly if you zoom in). The registration errors are small compared to the browning regions.

**Figure 3:**
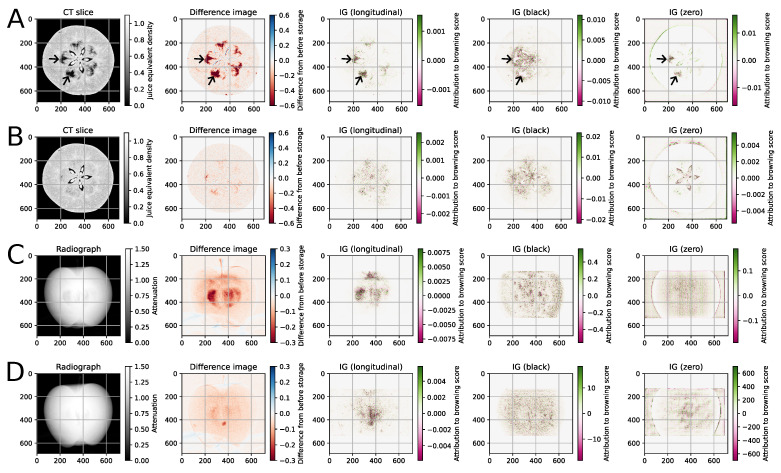
Examples of IG heatmaps with different baselines (longitudinal, black, or zero). (**A**) Result on the CT detection network. (**B**) Result on the CT prediction network. (**C**) Result on the radiograph detection network. (**D**) Result on the radiograph prediction network. All images were calculated on the same apple with browning score 4. IG with a black baseline is unable to highlight black regions, which explains why the two spots marked with arrows are not highlighted, even though they are strong indicators of browning. IG with a zero baseline highlights the background, even though this region is irrelevant in detecting browning.

Figure 2 shows an example of CT scans and simulated radiographs after image registration. The changes between the before-storage and later scans are shown as difference images. Some difference images show registration artifacts around the edges of the apple or the apple core, indicating some registration error. However, the registration error is very small compared to the features of interest. A visual inspection of the whole dataset showed registration errors in a similar range as visible in Figure 2 and Figure 3.

Table 5 shows the (average) outputs of the neural networks on the different baselines. On healthy apples, the average output of the longitudinal baseline is close to one, so it can be considered a neutral baseline. For brown apples, outputs on the longitudinal baseline are slightly higher. Both the black and the zero baselines have outputs that are far outside of the 1–4 range on at least one network. In those cases, most of the intensity in the IG heatmaps explains the baseline rather than the input.

Figure 3 shows examples of IG heatmaps with the longitudinal, black, and zero baselines for all four networks. It shows that the heatmap with a black baseline (fourth column) on the CT detection network (first row) can not highlight the severely brown regions because they have the same intensity on the CT slice as on the baseline. Additional examples are included in Appendix A.

## 5. Discussion

### 5.1. Findings on the ‘Braeburn’ Browning Dataset

Our results in Table 3 align with existing research indicating that it is possible to detect browning after a shelf-life period from CT slices or radiographs [4] and to distinguish 4 classes of browning severity [23]. To the knowledge of the authors, there is no previous research on predicting browning from just-out-of-CA-storage CT slices or radiographs, making this the most promising finding. However, the sensitivity of developing browning in ‘Braeburn’ apples depends on the orchard [72] and weather conditions [29], but the ‘Braeburn’ browning dataset in this paper only contains data from a single orchard and a single growing season. Moreover, although the dataset contains a large number of CT slices and radiographs, they were acquired from a relatively small number of apples (Table 2). Therefore, while our our results on predicting internal browning are promising, experiments on additional data are required to verify their general applicability.

The worse MAE and accuracy on the radiography networks compared to the CT slice networks can be explained by the higher contrast of the CT slices. The CT slices also focused only on the center of the apple, which shows the most changes due to core browning. Combining the results from multiple images improved the results slightly. Earlier work [23] also suggested that a single CT slice is sufficient for detecting browning.

The detection and prediction networks presented here do not generalize to each other’s data (Table 4). This is likely because the regions of the CT slice around the core that are associated with browning get darker during shelf-life (Figure 2), so the detection networks have been trained to detect darker spots than the prediction networks. Example images of the input data of the detection and prediction networks at each browning score are provided in Appendix A. These results suggest that, for grading browning, it is important to consider the moment of data acquisition relative to the progression of the disorder and the moment of consuming the apple.

All four networks showed higher average browning scores on the before-storage (longitudinal) baseline for apples that became brown after CA storage and a shelf-life period (Table 5). While this means the baseline is less neutral, it also suggests that the neural networks are using some information from the before-storage images to detect or predict browning, which can be considered a partial explanation. Moreover, these results suggest that it might be possible to use deep learning to identify apples before CA storage that would be predisposed to developing internal browning during (non-optimal) CA storage. This may be an interesting subject for future research.

### 5.2. Methodology of the Workflows

In this paper, the browning scores were modeled as a single continuous variable, so the neural networks used an image regression approach. It would also have been possible to model the browning scores as four separate classes, resulting in an image classification approach. Many XAI papers show examples on image classification but not on image regression [17,19,73]. However, for ordered classes such as the browning score, the regression approach used in this paper has the advantage that a single heatmap can explain the entire network. In the image classification case, a heatmap would be calculated for each class. Moreover, the outputs of image classification networks are constrained to sum to 1, so the network can only have a high output for one class. This would make it challenging to compare heatmaps of apples that were classified at different scores. There are also ordinal regression approaches [74,75] that maintain the discrete nature of the classes, but take into account their ordering. However, like image classification, these methods use multiple outputs, so the results on one image can not be explained by a single heatmap.

The main aim of this study was to demonstrate the two workflows. Therefore, we used only one network architecture (EfficientNetV2_s), and did not include an evaluation of data augmentation techniques and their hyperparameters. For use in industrial practice or for research on the optimal detection of specific disorders, we recommend evaluating multiple network architectures and data augmentation approaches.

The IG XAI method [17] was used in workflow 2 because it is commonly used and has been implemented in several explainable AI libraries, such as Captum and Saliency. The paper on IG also introduced the axiom-based approach of calculating XAI heatmaps. Recently, several similar methods for calculating heatmaps have been proposed, with the aim of producing results that are less visually noisy. Several of these methods also require a baseline and satisfy the completeness axiom [19,44,47,48]. Therefore, it would be interesting future work to combine workflow 2 with these methods, as this may lead to less visually noisy heatmaps. The results from Table 5 only rely on evaluating the neural network at the baseline and input images, and on the completeness axiom, so they directly apply to these methods as well. Moreover, these methods also share the property that they are blind to areas that are the same between the input and the baseline.

### 5.3. Considerations for Applications in Practice

Acquisition time is a major constraint in real-world sorting systems. Commercial sorting systems have a throughput of roughly 10 apples per second, corresponding to a maximum acquisition time of 100 ms per sample. The radiographs used in this paper were acquired in 100 ms, so it is likely that a similar radiography-based system could be implemented in practice. The CT slices had a significantly longer acquisition time but higher accuracy, so the results on CT slices can serve as an indicator of the performance that is possible when there is no limit on acquisition time. The scanning setup used in this paper was not optimized for speed. Therefore, using optimized hardware is likely to yield similar performance with shorter acquisition times for both radiographs and CT slices. Research is ongoing to increase the throughput of CT scanners [76,77,78], so real-time CT scanning might become available in the future.

The prediction network in this paper predicted browning after a fixed two-week shelf-life period. However, by collecting a dataset with different shelf-life periods, it would be possible to train neural networks with multiple outputs, each for a different shelf-life period. This could be used to estimate best-before dates for each product individually. Such an approach could also be valuable in predicting ripeness in fruit with a narrow ripeness window, such as mangoes or avocados. Moreover, it might be possible to improve the estimations by providing the duration of CA storage as an additional input to the neural network.

Longitudinal XAI Heatmaps can also be applied to other imaging modalities, such as MRI or photography, and problems outside of the postharvest domain [54]. The main requirement is that the images are precisely aligned. This alignment may be achieved through image registration, as proposed in this paper, making the method more widely applicable. One example from medicine would be to extend the study of Wang et al. [79] on explaining networks for predicting a person’s age from an MRI scan of their brain, which was done on the longitudinal Rotterdam study dataset. An image registration-based longitudinal baseline could be used to limit the explanation to changes occurring after a certain age. Moreover, it may reduce artifacts compared to other baselines, such as the black or zero baseline, which are also outside the data distribution for brain MRI images.

## 6. Conclusions

This paper presented two workflows for using longitudinal CT datasets in the development of X-ray-based postharvest disorder detection systems, and demonstrated them on a dataset of ‘Braeburn’ apples developing internal browning. The first workflow can be used to compare the detection accuracy at different time points within the development of the disorder, and to test whether networks generalize over time. On the ‘Braeburn’ browning dataset, the browning score could be detected after a shelf-life period or predicted from just-out-of-CA-storage data using deep learning. However, these neural networks did not generalize to each other’s data, indicating that it is important to consider the time point of image acquisition relative to the development of a disorder. The second workflow can be used to generate XAI heatmaps that only explain changes over time. On the ‘Braeburn’ browning dataset, this resulted in heatmaps that were not blind to regions of severe browning and did not exhibit severe activations in the background. While a more varied dataset is required to confirm our findings on the prediction of browning in ‘Braeburn’ apples, the results in this paper are a promising step towards developing real-world, explainable early detection systems for this disorder. Moreover, the workflows in this paper are sufficiently general to be applied to other 3D imaging modalities and to other gradually developing disorders, even outside the postharvest domain, which may benefit a wide range of future research.

## Figures and Tables

**Table 1:** Overview of which apples were used for the detection and prediction tasks.

		9 Weeks CA		12 Weeks CA	17 Weeks CA	
		14 Days Shelf-Life	21 Days Shelf-Life	14 Days Shelf-Life	7 Days Shelf-Life	14 Days Shelf-Life
Detection	Healthy	4	1	15	5	3
	Brown	1	2	12	15	17
Prediction	Healthy	3	N.A.	15	N.A.	3
	Brown	1	N.A.	12	N.A.	17

**Table 2:** Division of apples into the train, validation, and test sets and the resulting number of CT slices and radiographs.

	Train Set			Validation Set			Test Set		
	Apples	CT Slices	Radiographs	Apples	CT Slices	Radiographs	Apples	CT Slices	Radiographs
Detection	45	5550	21,600	15	1848	7200	15	1884	7200
Prediction	31	3798	14,880	10	1240	4800	10	1248	4800

**Table 3:** Results of the neural networks on the test-set.

		Browning Score MAE		Brown/Healthy Accuracy	
		Per-Image	Per-Apple	Per-Image	Per-Apple
CT slices	Detection	0.33	0.20	96.7%	100%
	Prediction	0.19	0.00	90.6%	100%
Radiographs	Detection	0.43	0.40	85.7%	86.7%
	Prediction	0.63	0.60	78.6%	80.0%

**Table 4:** Results of the neural networks when they were applied to data from a different time point than what they were trained on.

		Browning Score MAE (Per-Image)	% Classified as Score 1	% Classified as Score 4
CT Slices	Detection network on just-out-of-CA-storage data	1.04	85.4%	0.0%
	Prediction network on after-shelf-life data	1.56	1.4%	87.8%
Radiographs	Detection network on just-out-of-CA-storage data	1.09	67.6%	0.0%
	Prediction network on after-shelf-life data	1.21	4.0%	60.4%

**Table 5:** Neural network output for different baseline choices.

		Longitudinal on Healthy Apples	Longitudinal on Brown Apples	Black	Zero
CT Slices	Detection	1.00±0.03	1.12±0.23	1.12	−13.64
	Prediction	1.07±0.07	1.55±0.30	1.18	1.14
Radiographs	Detection	1.14±0.20	1.54±0.36	1.38	614.50
	Prediction	1.29±0.05	1.29±0.07	826.45	46,903.21

**Figure A1:**
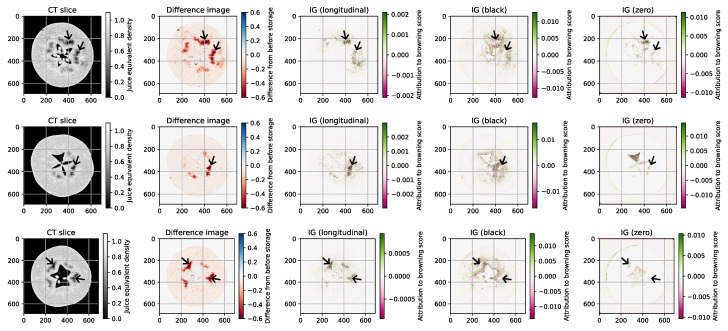
Additional examples of IG heatmaps with different baselines (longitudinal, black, or zero) on the CT detection network. IG with a black baseline is unable to highlight black regions, which explains why the spots marked with arrows are not highlighted, even though they are strong indicators of browning.

**Figure A2:**
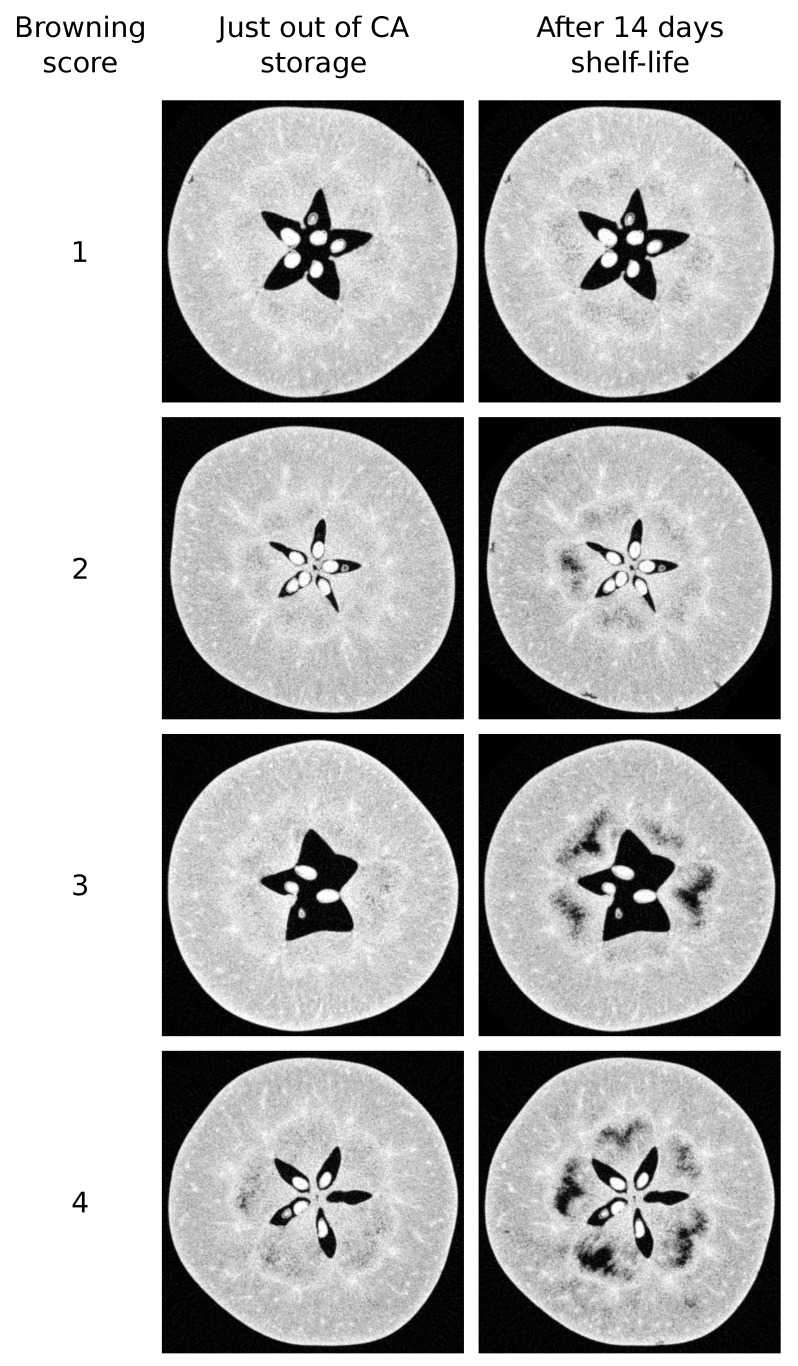
Example CT slices at different browning scores and scanning time points. The *Just out of CA storage* images on the left are used as input to the prediction networks, and the *After 14 days shelf-life* images on the right are used as input to the detection networks. The dark spots around the core that are associated with browning are a lot darker on the *After 14 days shelf-life* images than on the *Just out of CA storage* images. This difference explains why the detection and prediction networks did not generalize to each other’s data (Table 4).

## Data Availability

The data presented in this study are available on request from the corresponding author. The large size of the dataset is the reason for not publishing the data alongside the paper. All computational methods were implemented in Python (version 3.11), and the code is publicly available on Github (https://github.com/D1rk123/longitudinal_ct_workflow, accessed on 6 July 2026).
